# The impact of adjuvant radiotherapy on borderline and malignant phyllodes tumors of the breast

**DOI:** 10.1007/s12282-025-01725-3

**Published:** 2025-05-22

**Authors:** Amonthep Charoenyothakun, Kanjana Shotelersuk, Chonnipa Nantavithya, Kitwadee Saksornchai

**Affiliations:** 1 Division of Radiation Oncology, Department of Radiology, King Chulalongkorn Memorial Hospital, Thai Red Cross Society, Bangkok, Thailand; 2 Division of Radiation Oncology, Faculty of Medicine, Chulalongkorn University, Bangkok, 10330 Thailand

**Keywords:** Phyllodes tumor, Radiotherapy, Breast

## Abstract

**Background:**

Borderline and malignant phyllodes tumors (PTs) are rare fibroepithelial breast neoplasms associated with a high risk of locoregional recurrence (LRR). Although adjuvant radiation therapy (RT) is increasingly used, its clinical benefit remains uncertain. This study aimed to assess the impact of RT and identify factors associated with LRR in patients with borderline and malignant PTs.

**Methods:**

A retrospective review was conducted on 102 patients (50 borderline, 52 malignant PTs) who underwent surgery between 2012 and 2021. Clinical, pathological, and treatment data were analyzed. The primary endpoint was LRR. Kaplan–Meier and Cox regression models were used to assess recurrence and risk factors.

**Results:**

Median follow-up was 4.3 years. Malignant PTs were more likely to be > 10 cm (63.5% vs. 22%), undergo mastectomy (75% vs. 11%), and receive adjuvant RT (78.9% vs. 8%) compared to borderline PTs (all P < 0.001). Among patients without RT, malignant PTs had a significantly higher LRR than borderline PTs (36.4% vs. 4.4%, P < 0.010). In malignant PTs, RT was associated with a lower LRR (12.2% vs. 36.4%), though not statistically significant (P = 0.081). Tumor subtype was the only independent predictor of LRR (P = 0.011). Among malignant PTs who received RT, treatment initiation beyond 12 weeks post-surgery was associated with increased LRR (P = 0.009). Radiation technique, dose, and use of bolus were not significantly associated with LRR.

**Conclusion:**

Malignant PTs demonstrated higher LRR than borderline PTs. While the benefit of RT was not statistically significant, a trend toward reduced recurrence was observed.

**Supplementary Information:**

The online version contains supplementary material available at 10.1007/s12282-025-01725-3.

## Introduction

Phyllodes tumors (PTs) are rare fibroepithelial lesions that account for 0.3–1% of breast neoplasms. They are distinguished by enhanced stromal proliferation and “leaf-like” tumor cell growth patterns. The World Health Organization (WHO) guideline classifies PTs into benign, borderline, and malignant subtypes based on histopathologic characteristics [1, 2]. The malignant PTs account for 20% of all PTs and have a higher tendency to behave aggressively and metastasis [3]. Breast-conserving surgery (BCS) or total mastectomy (TM) with surgical margins of ≥ 1 cm are the mainstays of the curative treatment of PTs [4, 5]. According to recent reports of borderline and malignant PTs, the local recurrence rate (LRR) within 2 years after surgery is 21% and 36%, and distant metastases (DM) are 25% and 40%, respectively [5, 6]. The most common sites of metastasis are the lungs (66%), bone (28%), and brain (9%). Despite the generally favorable prognosis of PTs, the 5-year cancer-specific survival rates vary by subtype, ranging from approximately 80–92% for borderline and malignant PTs, with malignant subtypes exhibiting a higher risk of recurrence and metastasis.[7, 8].

Due to the high LR rates observed in borderline and malignant PTs, adjuvant RT has been increasingly used to enhance local control [7, 9]. While surgery remains the primary treatment, the role of RT in reducing recurrence risk has been explored in several studies, with varying conclusions. Barth et al.'s study, the only prospective study to date, reports excellent local control (LC) for borderline and malignant PTs treated with margin-negative BCS and adjuvant RT [5]. Gnerlich et al., an analysis of cases collected from NCDB from 1998 to 2009, adjuvant RT increased time to LR and a significant decrease in LR in women who received adjuvant RT versus surgery alone for malignant PTs but without a significant improvement in disease-free survival (DFS) or overall survival (OS) [7]. Belkacemi et al., demonstrated that adjuvant RT resulted in a higher 10-year LC rate for borderline and malignant PTs (86% with radiation versus 59% without radiation) [6]. Several retrospective studies have shown an improvement in LC; however, results are inconclusive and the indications for adjuvant local therapies remain debatable. Due to the rarity of the disease entity and especially the small numbers of borderline and malignant subtypes that received adjuvant RT, there were no randomized clinical trials and no studies involving the radiation technique.

This study aims to assess the efficacy of radiotherapy in patients with borderline and malignant PTs, identify factors associated with increased risk of LR, define subgroups of patients that may potentially benefit from adjuvant RT, and collect data involving the radiation technique.

## Material and methods

The study provided a retrospective review of all patients with borderline and malignant PTs of the breast who underwent surgery (BCS or TM) between 2012 and 2021 at our institute. Patients were excluded if they had: (1) recurrent phyllodes tumors or distant metastasis at diagnosis, (2) a history of other malignancies before or after PTs diagnosis, (3) a follow-up duration of less than two years, (4) prior radiation therapy to the breast, or (5) incomplete medical records preventing full data analysis. After IRB approval (IRB No. 0061/66), the medical records were examined for clinical data, tumor characteristics, treatment factors, and follow-up status. The primary endpoint was LRR. Kaplan–Meier and Cox regression models were conducted to determine LRR and the risk factors correlated with an increased risk of LR. A P value < 0.05 was considered statistically significant.

## Results

### Patient Characteristics

The median follow-up was 4.3 years. 102 patients were analyzed; 50 patients had borderline, and 52 patients had malignant PTs. Key variables examined included age at diagnosis, tumor size, surgical interventions, margin status, adjuvant RT, radiation techniques, dosage, fractionation, and radiation timing after surgery, as well as follow-up duration.

The median age at diagnosis was similar across groups: total populations (48 years), borderline (46 years), and malignant (49 years), with no statistically significant difference (P = 0.053). The median tumor size was significantly larger in the malignant group (12 cm) compared to the borderline group (5.6 cm), with a P value of < 0.001. Tumor size < 10 cm was more common in the borderline group (78%), whereas tumor size > 15 cm was predominantly in the malignant group (34.6%). BCS was more common in the borderline group (78%), while TM was more frequent in the malignant group (75%); this difference was statistically significant (P < 0.001). Most patients had margins ≥ 1 cm (total: 80.4%); no significant difference was observed in margin status across the groups (P = 0.300). Adjuvant radiation was administered to 78.9% of patients in the malignant group compared to 8% in the borderline group, with a P-value of < 0.001, a significant difference. Radiation technique (3D vs. IMRT/VMAT), total radiation dose, and fractionation were not significantly different between groups. The median timing for radiation after surgery was slightly longer for the borderline group but not significantly different (P = 0.130). Median follow-up duration was significantly longer in the borderline group (5.0 years) compared to the malignant group (3.7 years), with a P value of 0.046. Details in Table 1.

**Table 1:** Characteristics of patient

	Total (N = 102)	Borderline (N = 50)	Malignant (N = 52)	P value
Age (years) at diagnosis, Median (IQR)	48 (40–55)	46 (37–53)	49 (42.5–58)	0.053
Tumor size (cm), Median (IQR)	8 (4.8–14.1)	5.6 (4.1–8.8)	12 (6.6–17.5)	< 0.001
Tumor size (cm), n (%)				< 0.001
< 10	58 (56.9)	39 (78)	19 (36.5)	
10–15	23 (22.6)	8 (16)	15 (28.9)	
> 15	21 (20.6)	3 (6)	18 (34.6)	
Surgery, n (%)				< 0.001
BCS	52 (51)	39 (78)	13 (25)	
TM	50 (49.0)	11 (22)	39 (75)	
Margin, n (%)				0.300
≥ 1 cm	82 (80.4)	40 (80)	42 (80.8)	
< 1 cm	15 (14.7)	9 (18)	6 (11.5)	
Positive	5 (4.9)	1 (2)	4 (7.7)	
Adjuvant radiation, n (%)	45 (44.1)	4 (8)	41 (78.9)	< 0.001
Radiation technique, n (%)				0.233
3D	34 (75.6)	4 (100)	30 (73.2)	
IMRT/VMAT	11 (24.4)	0 (0)	11 (26.8)	
Total dose (Gy), median (IQR)	60 (51–60)	55 (50–60)	60 (51–60)	0.289
Total dose (Gy), n (%)				0.560
50–59	13 (28.9)	2 (50)	11 (26.8)	
60–66	32 (71.1)	2 (50)	30 (73.2)	
Bolus (Fx), n (%)				0.800
0	24 (53.3)	3 (75)	21 (51.2)	
1–10	12 (26.7)	1 (25)	11 (26.8)	
> 10	9 (20)	0	9 (22.0)	
Dose/Fx (Gy/Fx), n (%)				0.650
2	43 (95.6)	4 (100)	39 (95.1)	
3	2 (4.4)	0 (0)	2 (4.9)	
Radiation timing after surgery (Wk) median ( IQR)	8 (4–8)	10 (8–14)	8 (4–8)	0.130
Year of follow-up, median (IQR)	4.3 (2.5–5.8)	5.0 (2.7–6.5)	3.7 (2.3–5.3)	0.046

*BCS* breast conserving surgery, *TM* total mastectomy, *3D* three dimension conformal radiotherapy, *IMRT/VMAT* intensity modulated radiation therapy/volumetric modulated arc therapy, *Fx* fractions

### Clinical outcomes

There was no significant difference in LR between different surgery types, with P-values of 0.443 for borderline cases and 0.674 for malignant cases. Regarding the margin status, patients with a margin less than 1 cm and a positive margin had a significantly higher LR in malignant patients (P = 0.044). Adjuvant RT did not result in a difference in LR for either borderline or malignant patients. However, there was a trend toward a reduction in the number of recurrence cases when adding radiotherapy to malignant cases (12.2% vs. 36.4% without RT, P = 0.081). There were no significant differences in recurrence rates between RT technique, total dose, bolus administration, and dose per fraction (Gy/Fx). Details in Table 2.

**Table 2:** Proportion of local recurrence rate by groups

	Total (N = 102)	Borderline (N = 50)	Malignant (N = 52)
Overall	11 (10.8)	2 (4)	9 (17.3)
Surgery, n (%)			
BCS	5/52 (9.6)	2/39 (5.1)	3/13 (23.1)
TM	6/50 (12)	0/11 (0)	6/39 (15.4)
P value	0.598	0.443	0.674
Margin, n (%)			
> 1 cm	7/82 (8.5)	2/40 (5)	5/42 (11.9)
< 1 cm	3/15 (20)	0/9 (9.1)	3/6 (50)
Positive	1/5 (10)	0/1 (0)	1/4(25)
P value	0.263	0.576	0.044
Treatment modality, n (%)			
BCS	3/40 (7.5)	2/37 (5.4)	1/3 (33.3)
BCS + RT	2/12 (16.7)	0/2 (0)	2/10 (20)
P value	0.325	0.736	0.631
TM	3/17 (17.7)	0/9 (0)	3/8 (37.5)
TM + RT	3/33 (9.1)	0/2 (0)	3/31 (9.7)
P value	0.396	NA	0.088
Adjuvant radiation, n (%)			
No	6/57 (10.5)	2/46 (4.4)	4/11 (36.4)
Yes	5/45 (11.1)	0/4 (0)	5/41 (12.2)
P value	0.925	0.670	0.081
Radiation technique, n (%)			
3D	2/34 (5.9)	0/4 (0)	2/30 (6.7)
IMRT/VMAT	3/11 (27.3)	–	3/11 (27.3)
P value	0.085	NA	0.110
Total dose (Gy), n (%)			
50–59	1/13 (7.7)	0/2 (0)	1/11 (9.1)
60–66	4/32 (12.5)	0/2 (0)	4/30 (13.3)
P value	0.642	NA	0.713
Bolus (Fx), n (%)			
0	2/24 (8.3)	0/3 (0)	2/21 (9.5)
1–10	2/12 (16.7)	0/1 (0)	2/11 (18.2)
> 10	1/9 (11.1)	–	1/9 (11.1)
P value	0.821	NA	0.824
Dose/Fx (Gy/Fx), n (%)			
2	5/43 (11.6)	0/4 (0)	5/39 (12.8)
3	0/2 (0)	-	0/2 (0)
P value	0.609	NA	0.589

*BCS* breast conserving surgery, *TM* total mastectomy, *RT* adjuvant radiation therapy, *3D* three dimension conformal radiotherapy, *IMRT/VMAT* intensity modulated radiation therapy/volumetric modulated arc therapy, *Fx* fractions

**Fig. 1:**
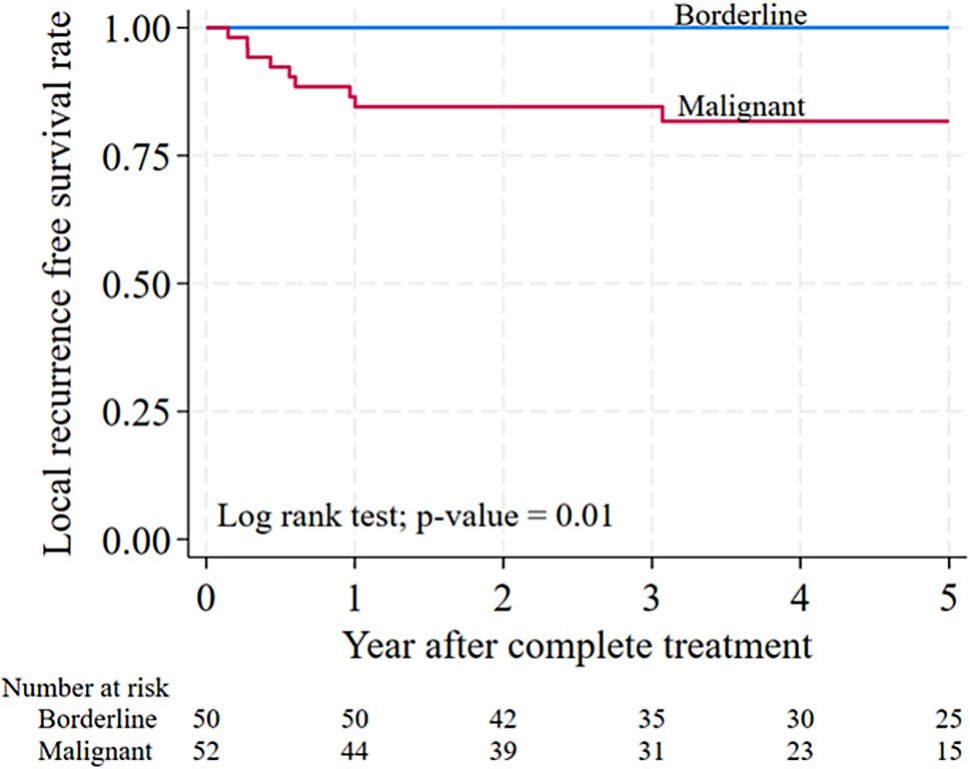
Cumulative of LRFS after surgery in borderline and malignant PTs

For patients with borderline and malignant PTs, the 5-year local recurrence-free survival rate (LRFS) after surgery was 100% and 81.7%, respectively. There was a statistically significant difference in LRFS after surgery between borderline and malignant patients (P = 0.010) (Fig. 1).

There was no statistically significant difference for 5-year LRFS after complete treatment (either surgery alone or surgery with RT) for all groups (BCS, BCS + RT, TM, and TM + RT were 97.5%, 83.3%, 81.5%, and 90.9%, respectively, P = 0.508) (Fig. 1S).

**Fig. 2:**
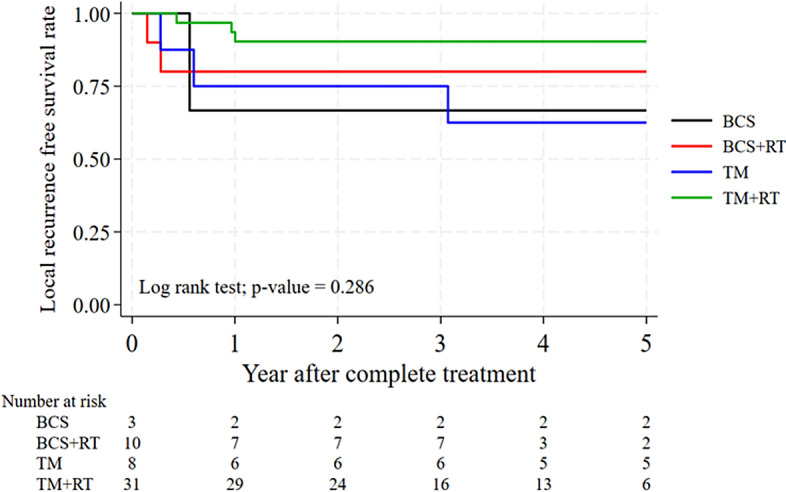
Cumulative of LRFS after surgery by treatment modality for malignant PTs

The 5-year LRFS for malignant patients was 66.7% with BCS alone, 80% with BCS + RT, 62.5% with TM alone, and 90.3% with TM + RT, with no statistically significant differences between these groups (P = 0.286) (Fig. 2).

### Prognostic factors

Our analysis revealed a significant association between tumor subtype and the risk of LR. Malignant PTs were associated with a much higher risk of LR compared to borderline PTs, with a hazard ratio (HR) of 5.7 (95% CI 1.22–27.36, P = 0.027) in the univariate analysis and an adjusted HR of 12.85 (95% CI 1.81–91.07, P = 0.011) in the multivariate analysis. Age at diagnosis did not significantly affect risk of LR, with patients over 50 showing no increased risk compared to those 50 or younger (HR 1.54, 95% CI 0.40–5.89, P = 0.525). Positive margins showed a trend toward higher recurrence, though this was not statistically significant. In terms of other factors, tumor size, surgery type (BCS vs. TM), and adjuvant RT did not demonstrate significant associations with the risk of LR in either univariate or multivariate analysis.

The timing of adjuvant RT was significantly associated with LR for malignant PT patients. Delaying RT beyond 12 weeks post-surgery resulted in a higher recurrence rate, with a hazard ratio (HR) of 18.9 (95% CI 2.09–170.6, P = 0.009) in the univariate analysis and an adjusted HR of 18.19 (95% CI 12.01–165, P = 0.010) in the multivariate analysis. RT technique, total dose, and bolus administration did not significantly influence local recurrence rates in our analysis. These findings were summarized in Tables 3, 1S and 2S.

**Table 3:** Risk factor associated with LR for malignant patient who received RT after surgery (N = 41)

	Univariate		Multivariate	
	HR (95% CI)	P value	aHR (95% CI)	P value
Age at diagnosis				
≤ 50	3.44 (0.38–30.81)	0.269		
> 50	1			
Tumor size (cm), group				
< 10	1			
≥ 10	0.65 (0.11–3.89)	0.637		
Surgery				
BCS	1			
TM	0.42 (0.07–2.53)	0.346		
Margin				
Negative	1			
Positive	1.17 (0.22–6.296)	0.425		
RT technique				
3D	1		1	
IMRT/VMAT	4.35 (0.72–26.1)	0.107	4.01 (0.66–24.21)	0.131
Total dose (Gy), n (%)				
50–59	1			
60–66	1.76 (0.20–15.78)	0.612		
Bolus (Fx)				
0	1			
≥ 1	1.57 (0.26–9.44)	0.617		
Radiation timing after surgery				
< 12 weeks	1		1	
≥ 12 weeks	18.9 (2.09–170.6)	0.009	18.19 (12.01–165)	0.010

Univariate and multivariate were evaluated with Cox regression model. Multivariate were developed by covariate with P < 0.2 from univariate

*HR* hazard ratio, *aHR* adjusted hazard ratio, *BCS* breast conserving surgery, *TM* total mastectomy, *RT* adjuvant radiation therapy, *3D* three dimension conformal radiotherapy, *IMRT/VMAT* intensity modulated radiation therapy/volumetric modulated arc therapy, *Fx* fractions

## Discussion

This retrospective analysis included a cohort of 102 patients, consisting of 50 with borderline PTs and 52 with malignant PTs, with a median follow-up duration of 4.3 years. Our patients’ characteristics were largely consistent with those reported in other studies, particularly the younger age group. The median tumor size in our study was larger than in other retrospective studies [10, 11], which median tumor sizes typically ranged from between 5 and 7.2 cm. Also, the proportion of malignant PTs (greater than 10 cm) was significantly higher compared to borderline PTs. This larger tumor size was associated with more aggressive surgical approaches, with a higher likelihood of TM for malignant cases. In our study, the majority of patients with malignant PTs underwent TM, compared to a much smaller proportion of borderline PT cases (75% vs. 11%, P < 0.001).

In our study, the 5-year LRFS rates were 100% for borderline phyllodes tumors (PTs) and 81.7% for malignant PTs. These rates were comparable to, and in some cases better than, those reported in other studies. For instance, Gnerlich et al. [7] reported 5-year LRFS rates of 94% for borderline PTs and approximately 75% for malignant PTs, indicating slightly lower rates for both groups compared to our findings.

The LRR in our study was lower than those reported in other studies, with 4% for borderline and 17.3% for malignant PTs. According to the WHO 2012 classification, LR can occur in any PT subtype, with an overall prevalence of 21%. The reported recurrence rates vary from 10 to 17% for benign PTs, 14–25% for borderline PTs, and 23–30% for malignant PTs [6, 11–13]. One possible explanation for the lower LRR in our study was the high rate of wide resection margins, with over 80% of cases having a margin greater than 1 cm. Although margin width was not a significant factor for LRR in our study, our results did show that close or positive surgical margins were associated with a higher LRR in malignant PTs. Other studies, such as those by Barrio et al. [14] and Tremblay-LeMay et al. [15], identified margin status as a key factor influencing local recurrence.

Several studies had shown the efficacy of adjuvant RT in reducing LRR in malignant PTs. For example, Barth et al. [5], the only prospective study to date, reported excellent LC for malignant PTs treated with margin-negative BCS and adjuvant RT, and a retrospective analysis by Gnerlich et al. [7] demonstrated that adjuvant RT significantly decreased LRR in patients with malignant PTs. In our study, we observed a trend toward lower local recurrence rates (LRR) in malignant PT patients who received adjuvant RT compared to those who did not (12.2% vs. 36.4%, P = 0.081), suggesting a potential benefit in selected high-risk cases. This result was consistent with other studies [5–7] and indicated the efficacy of adjuvant RT in reducing LRR, particularly in malignant PTs.

The timing of adjuvant RT administration post-surgery was a significant factor influencing LR risk in patients with malignant PTs in our study. Patients who received RT more than 12 weeks after surgery had a significantly higher LRR compared to those who received it earlier. To our knowledge, our study was one of the few to report on RT timing in phyllodes tumors as a potential factor influencing local control, emphasizing the importance of early initiation.

Other factors, such as patient age, tumor size, type of surgery, resection margin, and radiation technique or dose, did not demonstrate significant associations with LR risk in patients with borderline and malignant PTs. These findings were consistent with, but also differ in some respects from, other studies: Patient age, Macdonald et al. [8] similarly reported that age was not a significant predictor of recurrence in malignant PTs. However, some studies, such as Chaney et al. [16], suggested that younger age might be associated with higher recurrence rates, possibly due to biological differences in tumor behavior. Tumor size, Kapiris et al. [17] reported that tumor size had an effect on LC and survival in patients with malignant subtypes of PT, with a significantly increased risk for tumors larger than 10 cm. Type of Surgery and Resection Margin, Barrio et al. [12] also concluded that the choice of surgery (lumpectomy or mastectomy) was less critical than achieving negative surgical margins. NCCN [4] and The WHO classification [14] emphasized the importance of achieving negative margins to reduce recurrence rates.

Regarding RT technique and dose, our study found no significant differences in LRR between 3D and IMRT/VMAT, or between radiation doses, which was consistent with previous findings by Belkacemi et al. [6] and Gnerlich et al. [7]. However, it is important to acknowledge that selection bias may have influenced these findings, as higher-risk patients, such as those with larger tumors or close margins, may have been more likely to receive IMRT or higher radiation doses. This may have masked differences in efficacy between techniques or dose levels, and future prospective studies are needed to further clarify these associations.

### Limitations

The limitation of our study was the relatively small sample size, which may have limited the broader applicability of our findings. However, we had detailed information on the radiation techniques used, allowing for a more thorough analysis of the impact of adjuvant RT on LR in our study.

## Conclusion

Our study confirmed that malignant PTs had a higher LR rate compared to borderline PTs. While adjuvant RT did not significantly reduce LR, a trend toward improved local control was observed in malignant cases, suggesting a potential benefit in selected high-risk patients, particularly those with positive margins or large tumors.

Additionally, delayed RT was associated with an increased risk of recurrence, indicating that early initiation of RT might have been beneficial for patients undergoing adjuvant treatment. However, further research was needed to better define the optimal timing and patient selection criteria for RT in this setting.

Given the lack of prospective trials in PTs, future research should have focused on establishing multicenter collaborations to improve data collection and treatment standardization. This study may also have contributed to defining the optimal timing of adjuvant radiotherapy, ensuring that treatment was initiated in a timely manner to maximize local control and reduce recurrence risk.

## Supplementary Information

Below is the link to the electronic supplementary material.

Supplementary file1 (DOCX 119 KB)
