# Supplementary material for: The impact of adjuvant radiotherapy on borderline and malignant phyllodes tumors of the breast
Source: Breast Cancer. 2025 May 22;32(5):1006–12. doi: 10.1007/s12282-025-01725-3 (PMC12394283; doi:10.1007/s12282-025-01725-3)
Supplement: Supplementary file 1 — Supplementary file1 (DOCX 119 KB) [file 12282_2025_1725_MOESM1_ESM.docx]

**Supplementary Appendix**

**Figure 1S** cumulative of LRFS after surgery by treatment modality


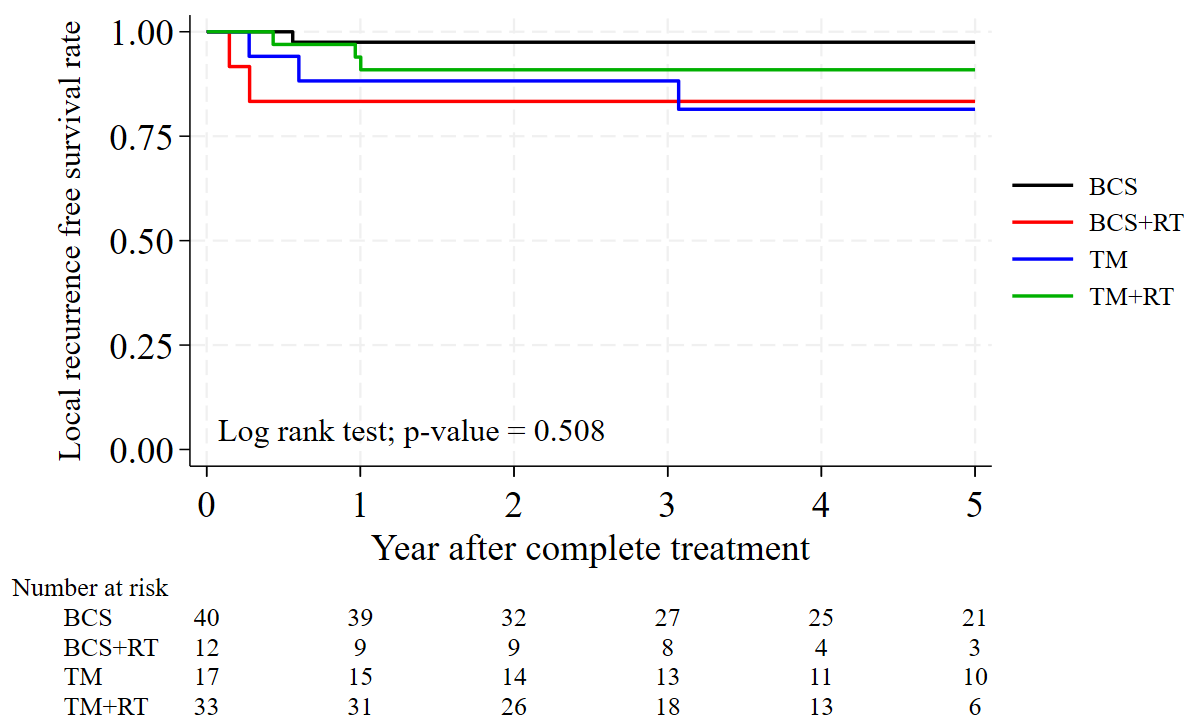


**Table 1S** Risk factor associated with LR for all patients

|  | Univariate | | Multivariate | |
| --- | --- | --- | --- | --- |
|  | HR (95% CI) | P-value | aHR (95% CI) | P-value |
| Subtypes |  |  |  |  |
| - Borderline | 1 |  |  |  |
| - Malignant | 5.7 (1.22-27.36) | 0.027 | 12.85 (1.81-91.07) | 0.011 |
| Age at diagnosis |  |  |  |  |
| - ≤ 50 | 1.54 (0.40-5.89) | 0.525 |  |  |
| - > 50 | 1 |  |  |  |
| Tumor size (cm), group |  |  |  |  |
| - < 10 | 1 |  |  |  |
| - 10-15 | 1.22 (0.30-4.90) | 0.778 |  |  |
| - >15 | 1.06 (0.21-5.31) | 0.945 |  |  |
| Surgery |  |  |  |  |
| - BCS | 1 |  |  |  |
| - TM | 1.42 (0.43-4.70) | 0.293 |  |  |
| Treatment |  |  |  |  |
| - BCS | 1 | Ref | 1 | Ref |
| - BCS+RT | 4.12 (0.57-29.66) | 0.159 | 0.54 (0.07-4.34) | 0.562 |
|  |  |  |  |  |
| - TM | 1 | Ref |  |  |
| - TM+RT | 0.53 (0.11-2.63) | 0.435 |  |  |
| RT received |  |  |  |  |
| - No | 1 |  |  |  |
| - Yes | 1.35 (0.40-4.55) | 0.626 |  |  |
| Margin  - > 1 cm | 1 | ref |  |  |
| - < 1 cm | 0.61 (0.077-4.75) | 0.635 |  |  |
| - Positive | 2.55 (0.31-20.51) | 0.380 |  |  |

Univariate and multivariate were evaluated with Cox regression model. Multivariate were developed by covariate with P< 0.2 from univariate.

Abbreviations :HR = Hazard ratio, aHR = adjusted Hazard ratio, BCS= breast conserving surgery, TM= total mastectomy, RT = Adjuvant radiation therapy, 3D= Three dimension conformal Radiotherapy , IMRT/VMAT= Intensity Modulated Radiation Therapy/Volumetric Modulated Arc Therapy, Fx = Fractions

**Table 2S** Risk factor associated with LR for malignant patient

|  | Univariate | |
| --- | --- | --- |
|  | HR (95% CI) | P-value |
| Age at diagnosis |  |  |
| - ≤ 50 | 1.68 (0.42-6.75) | 0.461 |
| - > 50 | 1 |  |
| Tumor size (cm), group |  |  |
| - < 10 | 1 |  |
| - ≥ 10 | 0.69 (0.18-2.57) | 0.581 |
| Surgery |  |  |
| - BCS | 1 |  |
| - TM | 0.60 (0.15-2.43) | 0.480 |
| Treatment |  |  |
| - BCS | 1 |  |
| - BCS+RT | 0.67 (0.06-7.41) | 0.744 |
| - TM | 1 | Ref |
| - TM+RT | 0.25 (0.05-1.23) | 0.088 |
| RT received |  |  |
| - No | 1 |  |
| - Yes | 0.32 (0.09-1.21) | 0.093 |
| Margin |  |  |
| - Negative | 1 |  |
| - Positive | 1.48 (0.18-11.96) | 0.709 |

Univariate and multivariate were evaluated with Cox regression model. Multivariate were developed by covariate with P< 0.2 from univariate.

Abbreviations :HR = Hazard ratio, aHR = adjusted Hazard ratio, BCS= breast conserving surgery, TM= total mastectomy, RT = Adjuvant radiation therapy, 3D= Three dimension conformal Radiotherapy , IMRT/VMAT= Intensity Modulated Radiation Therapy/Volumetric Modulated Arc Therapy, Fx = Fractions
